# Transurethral cystolitholapaxy with the AH-1 stone removal system for the treatment of bladder stones of variable size

**DOI:** 10.1186/s12894-015-0003-z

**Published:** 2015-02-21

**Authors:** Aihua Li, Chengdong Ji, Hui Wang, Genqiang Lang, Honghai Lu, Sikuan Liu, Weiwu Li, Binghui Zhang, Wei Fang

**Affiliations:** Department of Urology, Yangpu Hospital, School of Medicine, Tongji University, 450 Tengyue Road, Shanghai, 200090 China; Department of Urology, the 411th Hospital of PLA, Shanghai, 200081 China

**Keywords:** Bladder stone, Transurethral cystolitholapaxy, Endoscopic surgery, AH-1 stone removal system

## Abstract

**Background:**

The treatment of large volume bladder stones by current equipments continues to be a management problem in both developing and developed countries. AH-1 Stone Removal System (SRS) invented by us is primarily used to crush and retrieve bladder stones. This study evaluated the safety and efficiency of transurethral cystolitholapaxy with SRS for the treatment of bladder stones of variable size.

**Methods:**

SRS, which was invented by Aihua Li in 2007, composed by endoscope, continuous-flow component, a jaw for stone handling and retrieving, lithotripsy tube, handle, inner sheath and outer sheath. 112 patients with bladder stones were performed by transurethral cystolitholapaxy with SRS since 2008. We compare the surgical outcome to bladder stones of variable size, and evaluate the surgical efficiency and safety.

**Results:**

Characteristics of patients and stone removal time in variable size were evaluated. To patients with single stone, stone size was 1.35 ± 0.37 cm and the operating time was 5.50 ± 3.92 min in Group A. Stone size was 2.38 ± 0.32 cm and the operating time was 11.90 ± 9.91 min in Group B. Stone size was 3.30 ± 0.29 cm and the operating time was 21.92 ± 9.44 min in Group C. Stone size was 4.69 ± 0.86 cm and the operating time was 49.29 ± 30.47 min in Group D. The difference was statistically significant between the four groups. Among them, 74 (66.07%) patients accompanied with benign prostatic hyperplasia (BPH) were treated by transurethral resection of the prostate (TURP) simultaneously. Compared between the four groups, the difference of the TURP time was not statistically significant, P >0.05. No significant complication was found in the surgical procedure.

**Conclusions:**

Transurethral cystolitholapaxy with SRS appears to be increased rapidity of the procedure with decreased morbidity. It is a safe and efficient surgical management to bladder stones. This endoscopic surgery best fits the ethics principle of no injury; meanwhile, the accompanied BPH could be effectively treated by TURP simultaneously.

## Background

Bladder stones account for 5% of urinary stones in the developed countries but for more in the developing countries. Novel modifications of these treatment modalities have been used for bladder stones. Unfortunately, the treatment of large volume bladder stones continues to be a management problem in both developing and developed countries [1-9].

Transurethral cystolitholapaxy is probably the most common way to manage cystolithiasis. But the transurethral methods by the current lithotriptoscope, nephroscope, cystoscope, resctoscope or ureteroscope are plagued by long operative times, trauma to the bladder mucosa, and still have several serious deficiencies [4,10-13]. SRS is primarily used to fragment and retrieve bladder stones, and is a dedicated endoscopic device with multiple functions such as stabilizing stone, fragmenting stone, automatically collecting fragments, retrieving stone, washing out stone and continuous irrigation in cystolitholapaxy. The primary outcome compared with a control group with current device appeared that the surgical procedure was safe and efficient [10-12].

The retrospective study was on 112 cases of bladder stones treated by transurethral cystolitholapaxy with SRS in last 7 years. We compare the surgical outcome to bladder stones of variable size, and further evaluate the surgical efficiency and safety.

## Methods

### Patients

Between July 2008 and March 2014, all 112 cases of bladder stone in our department were treated by transurethral cystolitholapaxy with SRS, after informed consents were obtained from patients. Among them, 74 (66.07%) patients accompanied with BPH were treated by TURP simultaneously. Follow-up was performed in 3 month postoperatively. Patients were between the ages of 43 and 97 years old, with a mean age of 74.1. Of these 112 patients, 97 were male and 15 were female.

112 patients were divided into four groups by the stone size. 54 patients with stone size <2 cm were in Group A, 34 patients with stone size from 2 to 2.9 cm were in Group B, 15 patients with stone size from 3 to 3.9 cm were in Group C, and 9 patients with stone size ≥4 cm were in Group D and the largest stone was 6.4 cm. The 54 patients in Group A were further divided to two subgroups by the surgical management to stones. Group A1 consisted of 10 patients and the stones were directly extracted using the jaw without lithotripsy; Group A2 had 44 patients and lithotripsy should be performed first and then fragments were retrieved. In 71 cases of bladder stone accompanied with BPH, TURP was performed with transurethral cystolitholapaxy simultaneously.

All patients were evaluated by physical examination, ultrasonography, plain abdominal radiography, complete blood count and blood biochemistry. The patients accompanied with BPH were further evaluated by International Prostate Symptom Score, serum prostate-specific antigen level. Stone size was measured by plain abdominal radiography and prostate volume was measured by ultrasonography or CT scan. The used irrigation fluid was saline in cystolitholapaxy and mannitol in TURP as usual.

### Surgical instruments and techniques

These surgical procedures were performed in the lithotomy position under spinal anesthesia. Holmium laser was used to perform cystolithotripsy, and the power setting used of holmium laser was 2.6–3.5 J and 2.0–2.5 Hz. The procedures were as follows.

**Figure 1:**
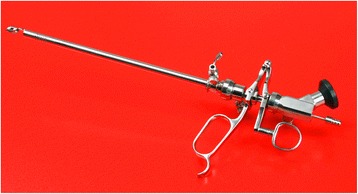
**AH-1 stone removal system (SRS).**

26 F SRS is composed by endoscope with illuminant and imaging component, continuous-flow component, a jaw for stone handling and stabilization during cystolithotripsy and stone retrieving during lithoextraction, lithotripsy tube, handle, inner sheath and outer sheath (Figure 1). Inner diameter of the outer sheath is 8.2 mm and it can be connected with Ellik evacuator. Sphere >60 mm in diameter can be stabilized with the jaw, sphere <15 mm can be grasped directly, and sphere <8 mm can be retrieved through outer sheath. The lithotripsy tube is 1.4 mm in inner diameter, by which, holmium laser fiber or pneumatic lithotripter probe can be passed to perform cystolithotripsy. The entire device is usually attached to a video camera to provide vision for the surgeon [10-12].

**Figure 2:**
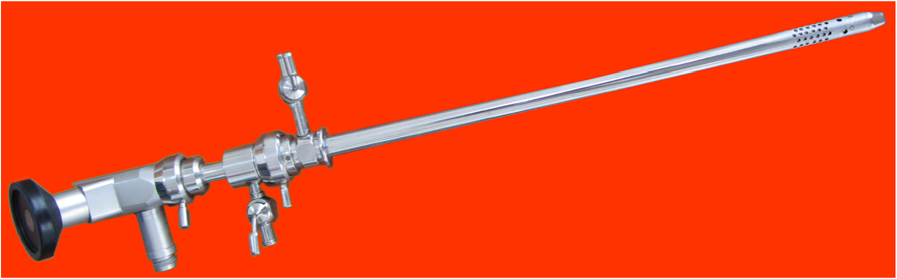
**The outer sheath with inner sheath and endoscope.**

**Figure 3:**
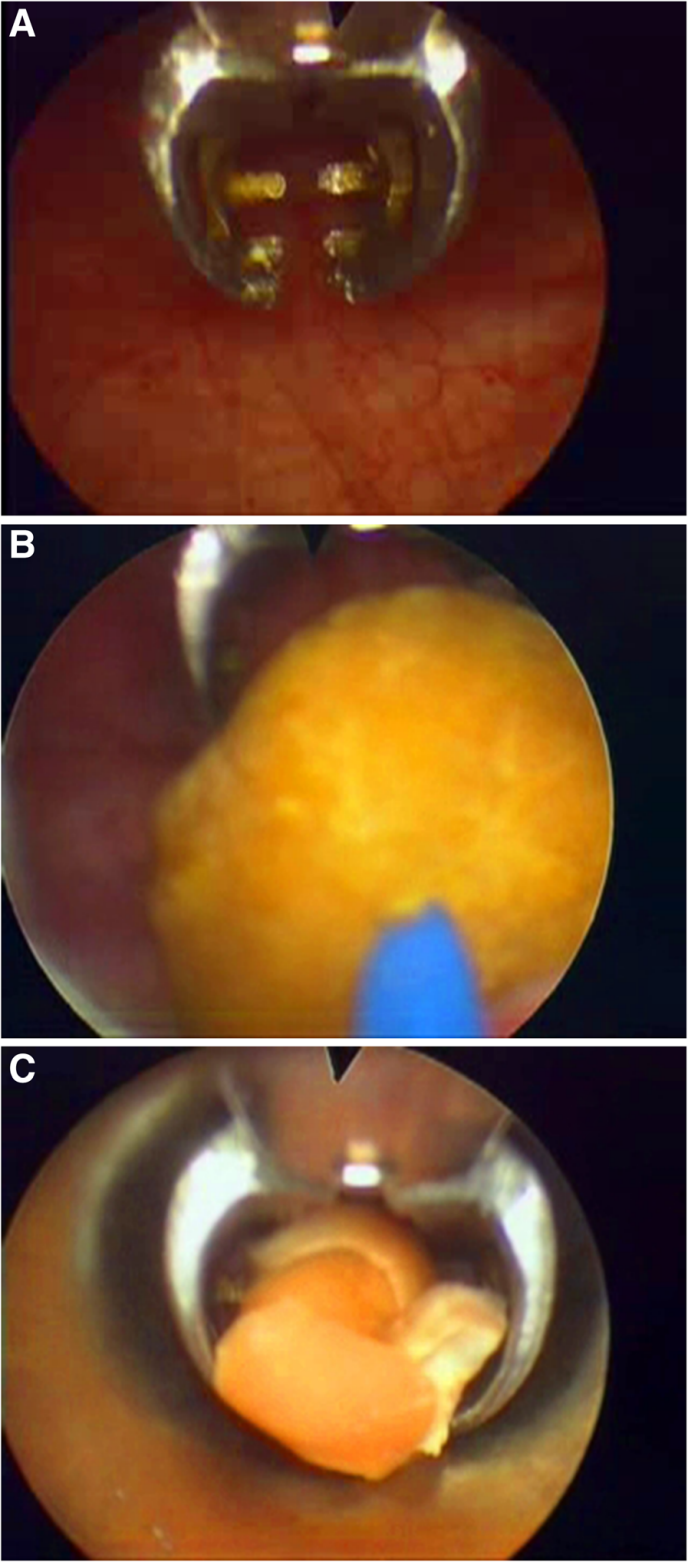
**Characteristics and functions of the jaw. A**. The jaw in endoscope; **B**. Stone was stabilized with the jaw and lithotripsy was performed with holmium laser; **C**. Fragments were retrieved using the jaw through outer sheath.

During the surgical procedure, first the outer sheath with inner sheath and endoscope was introduced into bladder in visual and conventional cystoscopy could be performed to check and visualize stones (Figure 2). Then, the inner sheath was removed and the working component was inserted into the outer sheath. By improving design, the working component also can go through a standard resectoscope sheath by a connector. After entering bladder and visualizing stones, stones were grasped and fixed using the jaw, and then cystolithotripsy was performed with holmium laser through lithotripsy tube (Figure 3B). After lithotripsy is completed, fragments could be retrieved using the jaw through outer sheath synchronously (Figure 3C). If there were more residual smaller fragments, Ellik evacuator could be further connected with the outer sheath and fragments were retrieved by Ellik evacuator. To patients accompanied with BPH, resectoscope was reinserted into urethra to perform TURP after cystolitholapaxy. A standard 26 F continuous-flow Storz resectoscope with a wing loop was used. The electrosurgical generator (Bircher Type-4400, USA) was set to 280–290 W of pure cutting current for the incision and 80–90 W for coagulation. Prostate tissue was resected to the surgical capsule of the prostate during the operating procedure. All surgical procedures were performed by one surgeon. The patients without other diseases were discharged within 48 hours and the patients simultaneously performed TURP or bladder neck incision was discharged in 5-7 days postoperatively [10-12].

SRS was designed by Aihua Li, M.D., and manufactured by Hangzhou Tonglu Shikonghou Medical Instrument Co., Ltd.

### Ethical consent

The study was approved by the Medical Ethics Committee of Yangou Hospital, School of Medicine, Tongji University.

### Statistical analysis

The differences of measurement data were compared using an unpaired *t* test and Chi-square test. Difference was considered significant at a P value <0.05. The reported values are the mean ± SD.

## Results

In all the 15 (13.39%) female patients, 4 (7.40%) cases were in Group A, 4 (11.76%) cases in Group B, 3 (20.00%) cases in Group C and 4 (44.44%) cases in Group D. Group D was compared with Group A, the difference was statistically significant, P <0.05. Stone size, stone number, patients performed by TURP simultaneously and the prostate volume in the four groups are shown in Table 1.

**Table 1:** **Characteristics of patients**

**Group**	**N**	**Stone size (cm)**	**Stone number**	**BPH**	**Prostate volume (ml)**
A1 (<2 cm)	10	0.84 ± 0.30	8.78 ± 8.45	9	44.87 ± 25.74
A2 (<2 cm)	44	1.43 ± 0.24	5.12 ± 6.36	31	56.93 ± 33.38
B (2-2.9 cm)	34	2.34 ± 0.31	2.62 ± 3.43	20	63.75 ± 42.71
C (3-3.9 cm)	15	3.27 ± 0.24	1.47 ± 1.20	10	63.12 ± 28.78
D (≥4 cm)	9	4.82 ± 0.83	2.44 ± 3.64	4	37.78 ± 18.04
Total	112			74	

Stone size was presented by the biggest in patients with multiple stones. Compared between the four groups, the difference of prostate volume was not statistically significant, P >0.05.

In Group A1, stones were directly retrieved by the jaw through the outer sheath or through urethra outside the sheath without lithotripsy. In Group A2 and other three groups, cystolithotripsy should be performed first and then fragments were retrieved by the jaw. In the surgery, stones <0.7 cm can be easily retrieved by the jaw through the outer sheath and more fragments could be retrieved by one extracting procedure. Stone removal time in different stone sizes and patients with single stone are shown in Tables 2 and 3.

**Table 2:** **Stone removal time in different stone sizes**

**Group**	**Lithotripsy**	**Stone removal time (min)**	**TURP time (min)**
A1 (<2 cm)	No	5.10 ± 2.13	32.50 ± 14.10
A2 (<2 cm)	Yes	11.11 ± 11.96	35.97 ± 14.92
B (2-2.9 cm)	Yes	17.30 ± 14.36	35.20 ± 10.74
C (3-3.9 cm)	Yes	20.68 ± 9.04	31.70 ± 12.44
D (≥4 cm)	Yes	64.11 ± 40.14	25.00 ± 12.25

Compared with Group A1, the difference of stone removal time was statistically significant, P <0.05 in Group B, P <0.001 in Group C and Group D. Compared with Group A2, the difference of stone removal time was statistically significant, P <0.01 in Group B and Group C, and P <0.001 in Group D. Compared with Group B, the difference of stone removal time was statistically significant, P <0.001 in Group D. Compared with Group C, the difference of stone removal time was statistically significant, P <0.01 in Group D. Compared between the four groups, the difference of TURP operating time was not statistically significant, P >0.05.

**Table 3:** **Stone removal time of patients with single stone**

**Group**	**N**	**Stone size**	**Stone removal time (min)**
A (<2 cm)	22	1.35 ± 0.37	5.50 ± 3.92
B (2-2.9 cm)	21	2.38 ± 0.32	11.90 ± 9.91
C (3-3.9 cm)	12	3.30 ± 0.29	21.92 ± 9.44
D (≥4 cm)	7	4.69 ± 0.86	49.29 ± 30.47

Compared with Group A, the difference of stone removal time was statistically significant, P <0.01 in Group B, P <0.001 in Group C and Group D. Compared with Group B, the difference was statistically significant, P <0.01 in Group C and P <0.001 in Group D. Compared with Group C, the difference was statistically significant, P <0.02 in Group D.

To 12 (10.71%) patients with BPH, the insertion of SRS was prevented by larger prostatic median lobe. So that TURP or bladder neck incision was first performed by F24 Storz resectoscope, to resect prostate or only the larger median lobe, and then cystolitholapaxy was performed. In this case, the surgical vision would be too blurred to perform surgery due to bleeding from resected fossa of prostate. In 4 (3.57%) patients with urethral stricture, urethral dilatation was performed first and then cystolithotripsy was done. But in another (0.89%) patients, the urethral stricture was too serious so that SRS was unable to be introduced into bladder. After SRS was further improved, the equipment can be introduced into the bladder under direct vision. And then the difficulty inserting into bladder was markedly improved, it appeared only in 3 patients (6.98%) among the later 43 patients.

No other significant complication was found in the surgical procedure and no patient was converted to an open procedure. The mean follow-up time was 35.18 ± 20.05 months (range 3-72 months) with no late complications related to the surgical procedure. None of the patients developed urethral stricture disease in the follow-up. All patients including whom with BPH and previously known urethral stricture disease had normal voiding function in the follow-up.

## Discussion

In the last decades, variable techniques for management of bladder stones have been mentioned in literature [1-12]. Open cystolithotomy, extracorporeal shockwave lithotripsy, percutaneous cystolitholapaxy and transurethral cystolitholapaxy are commonly performed in different medical center. The classical treatment for bladder stones still is transurethral cystolitholapaxy with lithotriptoscope, nephroscope, cystoscope, resctoscope or ureteroscope [1,2,4,10-14]. But the transurethral methods are either time consuming or have high morbidity, because four major deficiencies still can’t be perfectly resolved in the procedures [2]. First, bladder stone is easily rolling within bladder for the large cavity, which makes lithotripsy more difficult, especially to large volume or hard stone; Second, excessive fragments are produced after large bladder stone is crushed, and men urethra is slender and curl so that lithoextraction is not easy; Third, bladder wall is too thin to be easily damaged and ruptured in filling condition by irrigating solution [3,4]. Moreover, the current forceps used in endoscopic surgery can’t be used to stabilize stone during lithotripsy, only can be used to retrieve smaller stone and extract one fragment by one extracting procedure during lithoextraction [15]. Therefore, multiple entries to the urethra for lithoextraction would be needed, which will lead to urethral injury.

Bladder stones are rare in women [16,17]. In the study, patients with bladder stones in men were more than that in women and approximately 14% of all bladder stones occur in women, but with the stone size increasing, the proportion in women will be increased significantly.

In the surgical procedure, stones are stabilized with the jaw so that stones are no longer rolling in bladder, which makes lithotripsy more effective. Stones or fragments <7 mm can be easily retrieved by the jaw through the outer sheath. It effectively prevents multiple entries to the urethra and hence avoids possible urethral injury. The stones in 0.7-0.9 cm can be directly retrieved through urethra outside the sheath without lithotripsy, but the management will bring more injury to urethra so that it shouldn’t be used repeatedly. For retrieving smaller residual fragments, it’s also possible the use of a resectoscope connected to Ellik evacuator in order to avoid multiple entries to the urethra [10-12].

In the study, along with the increase of stone size, operating time is obviously increased. In 16 patients with BPH or urethral stricture, SRS was failure to be introduced into bladder, which was overcome by urethral dilatation, incision of the elevated bladder neck and TURP. Fortunately, after the structure of SRS was improved and the equipment can be introduced into the bladder under direct vision, the difficulty in insertion into the bladder was markedly improved [10-12].

About 15% patients were accompanied with bladder stones in the patients underwent TURP in our department. The surgical procedure can be safely combined with TURP [18-21]. We prefer to perform cystolitholapaxy first in the surgery, because surgical vision would be blurred after TURP, for associated bleeding from the resected fossa.

In the surgical procedure, working component should be introduced into bladder through the sheath in visual to avoid injury to urethra and bladder, after the sheath is inserted. Keeping low-pressure continuous irrigation and drainage to bladder during the surgical procedure is important to keep surgical vision clear and prevent bladder damage and rupture. For advanced aged and high risk patient, or too many, large and hard stones, the surgery could be performed in phases for surgical safety [10-12].

## Conclusions

Transurethral cystolitholapaxy by SRS appears to be increased rapidity of the procedure with decreased morbidity and is a safe and efficient surgery to bladder stones. The benefits of SRS apparently are the ability to grasp the stone to prevent moving whilst energy is being delivered to the stone and more rapid evacuation of crushed fragments. It also prevents multiple entries to the urethra and hence avoids possible urethral injury. It would be the better alternative for urologist to treat bladder stones. To the patients with urethral stricture and children, SRS in smaller size is needed to develop in the future [22,23].

## Abbreviations

BPH: Benign prostatic hyperplasia
SRS: AH-1 stone removal system
TURP: Transurethral resection of the prostate
